# Supplementary material for: In Vitro Antibacterial and Antibiofilm Activity of Hungarian Honeys against Respiratory Tract Bacteria
Source: Foods. 2021 Jul 14;10(7):1632. doi: 10.3390/foods10071632 (PMC8304482; doi:10.3390/foods10071632)
Supplement: Supplementary file 1 [file foods-10-01632-s001.zip › foods-1274319-supplementary.pdf]

Table S1. Statistical data of thin layer chromatography-direct bioautography (TLC-DB) assays

Haemophilus influenzae					
**One-way ANOVA**	**Sum of sqrs**	**df**	**Mean square**	**F**	**p-value**
Between groups:	208.724	3	69.574	232.600	6.878E-20
Within groups:	8.374	28	0.299		
Total:	217.098	31			
**Tukey's pairwise comparisons**					**p-value**
black locust honey *vs* positive control					<0.001
linden honey *vs* positive control					<0.001
sunflower honey *vs* positive control					<0.001
black locust honey *vs* linden honey					<0.01
black locust honey *vs* sunflower honey					>0.05
linden honey *vs* sunflower honey					>0.05
Haemophilus parainfluenzae					
**One-way ANOVA**	**Sum of sqrs**	**df**	**Mean square**	**F**	**p-value**
Between groups:	332.040	3	110.680	209.100	2.867E-19
Within groups:	14.819	28	0.529		
Total:	346.859	31			
**Tukey's pairwise comparisons**					**p-value**
black locust honey *vs* positive control					<0.001
linden honey *vs* positive control					<0.001
sunflower honey *vs* positive control					<0.001
black locust honey *vs* linden honey					<0.05
black locust honey *vs* sunflower honey					>0.05
linden honey *vs* sunflower honey					>0.05
Pseudomonas aeruginosa					
**One-way ANOVA**	**Sum of sqrs**	**df**	**Mean square**	**F**	**p-value**
Between groups:	113.951	3	37.983	413.700	2.779E-23
Within groups:	2.570	28	0.091		
Total:	116.522	31			
**Tukey's pairwise comparisons**					**p-value**
black locust honey *vs* positive control					<0.001
linden honey *vs* positive control					<0.001
sunflower honey *vs* positive control					<0.001
black locust honey *vs* linden honey					<0.01
black locust honey *vs* sunflower honey					>0.05
linden honey *vs* sunflower honey					>0.05
Streptococcus pneumoniae					
**One-way ANOVA**	**Sum of sqrs**	**df**	**Mean square**	**F**	**p-value**
Between groups:	225.752	3	75.250	217.000	1.747E-19
Within groups:	9.709	28	0.346		
Total:	235.461	31			
**Tukey's pairwise comparisons**					**p-value**
black locust honey *vs* positive control					<0.001
linden honey *vs* positive control					<0.001
sunflower honey *vs* positive control					<0.001
black locust honey *vs* linden honey					>0.05
black locust honey *vs* sunflower honey					<0.05
linden honey *vs* sunflower honey					<0.001

Table S2. Statistical data of agar well diffusion assays

***Haemophilus influenzae* 25%**

One-way ANOVA	Sum of sqrs	df	Mean square	F	p-value
Between groups:	19.615	4	4.903	117.500	7.96E-16
Within groups:	1.043	25	0.041		
Total:	20.658	29			

Tukey's pairwise comparisons	p-value
black locust honey 25% *vs* sugar solution 25%	<0.01
black locust honey 25% *vs* antibiotic control	<0.001
linden honey 25% *vs* sugar solution 25%	<0.001
linden honey 25% *vs* antibiotic control	<0.001
sunflower honey 25% *vs* sugar solution 25%	>0.05
sunflower honey 25% *vs* antibiotic control	<0.001
black locust honey 25% *vs* linden honey 25%	>0.05
black locust honey 25% *vs* sunflower honey 25%	>0.05
linden honey 25% *vs* sunflower honey 25%	>0.05
sugar solution 25% *vs* antibiotic control	<0.001

***Haemophilus influenzae* 50%**

One-way ANOVA	Sum of sqrs	df	Mean square	F	p-value
Between groups:	14.928	4	3.732	72.050	2.364E-13
Within groups:	1.295	25	0.051		
Total:	16.223	29			

Tukey's pairwise comparisons	p-value
black locust honey 50% *vs* sugar solution 50%	<0.05
black locust honey 50% *vs* antibiotic control	<0.001
linden honey 50% *vs* sugar solution 50%	<0.001
linden honey 50% *vs* antibiotic control	<0.001
sunflower honey 50% *vs* sugar solution 50%	<0.05
sunflower honey 50% *vs* antibiotic control	<0.001
black locust honey 50% *vs* linden honey 50%	>0.05
black locust honey 50% *vs* sunflower honey 50%	>0.05
linden honey 50% *vs* sunflower honey 50%	>0.05
sugar solution 50% *vs* antibiotic control	<0.001

***Haemophilus parainfluenzae* 25%**

One-way ANOVA	Sum of sqrs	df	Mean square	F	p-value
Between groups:	19.968	4	4.992	99.180	5.85E-15
Within groups:	1.258	25	0.050		
Total:	21.227	29			

Tukey's pairwise comparisons	
black locust honey 25% *vs* sugar solution 25%	<0.05
black locust honey 25% *vs* antibiotic control	<0.001
linden honey 25% *vs* sugar solution 25%	<0.001
linden honey 25% *vs* antibiotic control	<0.001
sunflower honey 25% *vs* sugar solution 25%	>0.01
sunflower honey 25% *vs* antibiotic control	<0.001
black locust honey 25% *vs* linden honey 25%	>0.05
black locust honey 25% *vs* sunflower honey 25%	>0.05
linden honey 25% *vs* sunflower honey 25%	>0.05
sugar solution 25% *vs* antibiotic control	<0.001

Haemophilus parainfluenzae 50%

One-way ANOVA	Sum of sqrs	df	Mean square	F	p-value
Between groups:	15.832	4	3.958	73.480	1.89E-13
Within groups:	1.346	25	0.053		
Total:	17.178	29			

Tukey's pairwise comparisons	p-value
black locust honey 50% *vs* sugar solution 50%	<0.01
black locust honey 50% *vs* antibiotic control	<0.001
linden honey 50% *vs* sugar solution 50%	<0.001
linden honey 50% *vs* antibiotic control	<0.001
sunflower honey 50% *vs* sugar solution 50%	<0.01
sunflower honey 50% *vs* antibiotic control	<0.001
black locust honey 50% *vs* linden honey 50%	>0.05
black locust honey 50% *vs* sunflower honey 50%	>0.05
linden honey 50% *vs* sunflower honey 50%	>0.05
sugar solution 50% *vs* antibiotic control	<0.001

Pseudomonas aeruginosa 25%

One-way ANOVA	Sum of sqrs	df	Mean square	F	p-value
Between groups:	26.335	4	6.583	298.400	1.06E-20
Within groups:	0.551	25	0.022		
Total:	26.887	29			

Tukey's pairwise comparisons	p-value
black locust honey 25% *vs* sugar solution 25%	>0.05
black locust honey 25% *vs* antibiotic control	<0.001
linden honey 25% *vs* sugar solution 25%	<0.001
linden honey 25% *vs* antibiotic control	<0.001
sunflower honey 25% *vs* sugar solution 25%	>0.05
sunflower honey 25% *vs* antibiotic control	<0.001
black locust honey 25% *vs* linden honey 25%	>0.05
black locust honey 25% *vs* sunflower honey 25%	>0.05
linden honey 25% *vs* sunflower honey 25%	<0.001
sugar solution 25% *vs* antibiotic control	<0.001

Pseudomonas aeruginosa 50%

One-way ANOVA	Sum of sqrs	df	Mean square	F	p-value
Between groups:	26.335	4	6.583	298.400	1.06E-20
Within groups:	0.551	25	0.022		
Total:	26.887	29			

Tukey's pairwise comparisons	p-value
black locust honey 50% *vs* sugar solution 50%	<0.01
black locust honey 50% *vs* antibiotic control	<0.001
linden honey 50% *vs* sugar solution 50%	<0.001
linden honey 50% *vs* antibiotic control	<0.001
sunflower honey 50% *vs* sugar solution 50%	<0.01
sunflower honey 50% *vs* antibiotic control	<0.001
black locust honey 50% *vs* linden honey 50%	>0.05
black locust honey 50% *vs* sunflower honey 50%	>0.05
linden honey 50% *vs* sunflower honey 50%	>0.05
sugar solution 50% *vs* antibiotic control	<0.001

Streptococcus pneumoniae 25%

One-way ANOVA	Sum of sqrs	df	Mean square	F	p-value
Between groups:	25.258	4	6.314	117.100	8.31E-16
Within groups:	1.348	25	0.053		
Total:	26.607	29			

Tukey's pairwise comparisons	p-value
black locust honey 25% *vs* sugar solution 25%	<0.001
black locust honey 25% *vs* antibiotic control	<0.001
linden honey 25% *vs* sugar solution 25%	<0.001
linden honey 25% *vs* antibiotic control	<0.001
sunflower honey 25% *vs* sugar solution 25%	<0.05
sunflower honey 25% *vs* antibiotic control	<0.001
black locust honey 25% *vs* linden honey 25%	>0.05
black locust honey 25% *vs* sunflower honey 25%	>0.05
linden honey 25% *vs* sunflower honey 25%	<0.05
sugar solution 25% *vs* antibiotic control	<0.001

Streptococcus pneumoniae 50%

One-way ANOVA	Sum of sqrs	df	Mean square	F	p-value
Between groups:	16.852	4	4.213	107.300	2.33E-15
Within groups:	0.981	25	0.039		
Total:	17.833	29			

Tukey's pairwise comparisons	p-value
black locust honey 50% *vs* sugar solution 50%	<0.001
black locust honey 50% *vs* antibiotic control	<0.001
linden honey 50% *vs* sugar solution 50%	<0.001
linden honey 50% *vs* antibiotic control	<0.001
sunflower honey 50% *vs* sugar solution 50%	<0.001
sunflower honey 50% *vs* antibiotic control	<0.001
black locust honey 50% *vs* linden honey 50%	>0.05
black locust honey 50% *vs* sunflower honey 50%	>0.05
linden honey 50% *vs* sunflower honey 50%	>0.05
sugar solution 50% *vs* antibiotic control	<0.001

Table S3. Statistical data of antibiofilm assays

Haemophilus influenzae					
**Mann-Whitney pairwise comparisons**					**p-value**
black locust honey *vs* linden honey					<0.001
black locust honey *vs* sunflower honey					<0.001
linden honey *vs* sunflower honey					<0.001
Haemophilus parainfluenzae					
**Mann-Whitney pairwise comparisons**					**p-value**
black locust honey *vs* linden honey					<0.001
black locust honey *vs* sunflower honey					<0.001
linden honey *vs* sunflower honey					<0.001
Pseudomonas aeruginosa					
**Mann-Whitney pairwise comparisons**					**p-value**
black locust honey *vs* linden honey					<0.001
black locust honey *vs* sunflower honey					<0.001
linden honey *vs* sunflower honey					<0.001
Streptococcus pneumoniae					
**One-way ANOVA**	**Sum of sqrs**	**df**	**Mean square**	**F**	**p-value**
Between groups:	2306.200	2	1153.100	34.560	1.98E-13
Within groups:	6005.490	180	33.363		
Total:	8311.690	182			
**Tukey's pairwise comparisons**					**p-value**
black locust honey *vs* linden honey					<0.001
black locust honey *vs* sunflower honey					>0.05
linden honey *vs* sunflower honey					<0.001
